# Unraveling olive germplasm diversity through integrated phenotypic and SSR-based characterization under temperate Himalayan conditions

**DOI:** 10.3389/fpls.2026.1934949

**Published:** 2026-10-27

**Authors:** Rizwana Rashid, Khalid Mushtaq Bhat, Abdul Raouf Malik, Mudasir Ahmad Mir, Munib-Ur- Rehman, Mohd. Amin Mir, Immad A. Shah, Ashaq Hussain Pandit, Javid Iqbal Mir, Khalid Ferooz, Tahya Bashir, Arshe Khurshid, Bodade Pranay Shrikrushna

**Affiliations:** 1 Division of Fruit Science, Faculty of Horticulture, Sher-e-Kashmir University of Agricultural Sciences and Technology of Kashmir, Srinagar, Jammu and Kashmir, India; 2 Division of Plant Biotechnology, Sher-e-Kashmir University of Agricultural Sciences and Technology of Kashmir, Srinagar, Jammu and Kashmir, India; 3 Division of Agricultural Statistics, Faculty of Horticulture, Sher-e-Kashmir University of Agricultural Sciences and Technology of Kashmir, Srinagar, Jammu and Kashmir, India

**Keywords:** germplasm characterization, Himalayan conditions, *Olea europaea*, phenotypic diversity, SSR markers

## Abstract

**Introduction:**

Olive (*Olea europaea* L.) cultivation is expanding beyond its traditional Mediterranean production regions; however, information on cultivar performance, phenotypic diversity, and molecular relationships under temperate Himalayan conditions remains limited. This study aimed to characterize olive germplasm using integrated morpho-pomological and simple sequence repeat (SSR) approaches.

**Methods:**

Thirteen olive accessions, comprising eleven named cultivars and two local selections (S1 and S2), were evaluated using 24 morpho-pomological descriptors of fruits, stones, and leaves. Analysis of variance, correlation analysis, principal component analysis, hierarchical clustering, and multiple regression were used to assess phenotypic variation and relationships. Molecular characterization was performed using three polymorphic SSR loci (UDO99-019, EMO3, and EMO88), and allele-presence profiles were analysed using Jaccard similarity and unweighted pair-group method with arithmetic mean (UPGMA) clustering.

**Results:**

Analysis of variance revealed highly significant (*P* ≤ 0.001) differences among accessions for all quantitative traits. Fruit weight ranged from 2.13 to 7.95 g, while the flesh-to-stone ratio varied from 2.16 to 6.06. Fruit weight was strongly correlated with fruit diameter (*r* = 0.859), flesh-to-stone ratio (*r* = 0.764), fruit length (*r* = 0.716), and stone weight (*r* = 0.699). The first two principal components explained 68.3% of the total phenotypic variation, with fruit size-related traits contributing most strongly to the multivariate structure. Hierarchical clustering separated the accessions into four phenotypic clusters. Multiple regression identified fruit length and flesh-to-stone ratio as significant positive predictors of fruit weight, whereas leaf length showed a significant negative conditional association. The three SSR loci generated 11 alleles, averaging 3.67 alleles per locus, with polymorphism information content (PIC) values ranging from 0.31 to 0.43 (mean 0.36), indicating moderate marker-level diversity. UPGMA clustering separated the accessions into three major groups based on Jaccard similarity of SSR allele-presence profiles.

**Discussion:**

S1, S2, and Carolea exhibited comparatively favourable fruit characteristics, including higher fruit weight, flesh-to-stone ratio, and desirable fruit dimensions. The integrated morpho-pomological and SSR approaches provided complementary information for phenotypic characterization, cultivar discrimination, and germplasm documentation, establishing a baseline for further multi-environment and higher-resolution molecular evaluation of olive germplasm under Himalayan conditions.

## 1 Introduction

Olive (*Olea europaea* L.) is one of the oldest domesticated fruit tree species and among the most economically important perennial crops of the Mediterranean basin. Belonging to the family Oleaceae, olive originated in the eastern Mediterranean and Upper Mesopotamian regions and subsequently spread throughout southern Europe, North Africa, and western Asia before being introduced into diverse agroecological zones worldwide (Ozkaya et al., 2006; Heywood et al., 2007). At present, olive is cultivated in more than 54 countries over an estimated area exceeding 11 million hectares, reflecting its global economic importance as both an oil and table fruit crop (Besnard et al., 2018). Olive oil is highly valued for its elevated content of monounsaturated fatty acids, particularly oleic acid, together with phenolic compounds and other bioactive constituents associated with numerous nutritional and health-promoting properties (Kalaitzis and El-Zein, 2016; Guclu et al., 2021).

The long history of olive cultivation, coupled with centuries of cross-pollination, vegetative propagation, natural mutation, and farmer-mediated selection, has resulted in remarkable genetic and phenotypic diversity. More than 1,200 cultivars have been documented worldwide, with the highest concentration occurring within the Mediterranean region (Bartolini et al., 2005; Belaj et al., 2016). This diversity represents a valuable reservoir of genetic resources for breeding programs targeting improved fruit quality, oil yield, productivity, climate resilience, and adaptation to emerging production environments. However, effective conservation and utilization of olive germplasm remain challenging due to extensive synonymy, homonymy, and cultivar misidentification, often exacerbated by environmental influences on phenotypic traits (Gomes et al., 2018).

Morphological and pomological characterization remains the cornerstone of olive germplasm evaluation and cultivar description. Standardized descriptors encompassing vegetative growth, fruit morphology, and stone characteristics are widely employed in germplasm management, breeding, and cultivar registration programs (Barranco et al., 2000; Arias-Calderon et al., 2014; Casilla García et al., 2025). Traits such as fruit weight, fruit dimensions, flesh-to-stone ratio, and stone morphology directly influence consumer preference, processing suitability, and commercial value. Significant variation in these descriptors has been reported among olive cultivars, highlighting their utility in cultivar discrimination and selection of superior genotypes (Khadivi et al., 2022; Dehghan-Seresht et al., 2025).

Despite their practical value, morphological descriptors alone are often insufficient for accurately inferring genetic relationships because trait expression may be influenced by environmental conditions, orchard management practices, tree age, and developmental stage (Rotondi et al., 2003; Rao et al., 2009). Consequently, phenotypically similar cultivars may differ genetically, while genetically related cultivars may exhibit contrasting morphological characteristics under different environments. Integrating phenotypic characterization with molecular approaches therefore provides a more robust framework for cultivar identification, germplasm management, and diversity assessment.

In recent years, olive cultivation has expanded beyond its traditional Mediterranean distribution into non-conventional production regions, including the north-western Himalayan belt of India. The temperate regions of Jammu and Kashmir possess favorable climatic conditions characterized by adequate winter chilling, moderate summer temperatures, and suitable altitude gradients that support successful olive cultivation (Lal et al., 2016). Government-led initiatives promoting crop diversification, orchard establishment, and nursery development have further accelerated the introduction and commercialization of olive cultivation in the region (Sharma et al., 2023). Although several studies have evaluated the growth, yield performance, and variability of olive cultivars under mid-hill conditions of Jammu and Kashmir, information regarding the integrated phenotypic and molecular characterization of olive germplasm cultivated under the unique environmental conditions of the North-Western Himalayas remains scarce (Kumar et al., 2020, 2021). This knowledge gap is particularly important because environmental heterogeneity, altitude-associated climatic variation, and localized adaptation may influence cultivar performance and genetic structure, thereby affecting cultivar recommendation, breeding strategies, and long-term germplasm conservation. Furthermore, the absence of molecular fingerprints for olive cultivars cultivated in the region limits accurate cultivar authentication and the establishment of region-specific germplasm databases.

Among available molecular marker systems, simple sequence repeats (SSRs) are considered one of the most informative tools for olive genetic studies owing to their high polymorphism, co-dominant inheritance, reproducibility, and effectiveness in detecting genetic variation and cultivar-specific alleles (Belaj et al., 2003; Koehmstedt et al., 2011). SSR markers have been extensively applied in olive germplasm characterization, cultivar authentication, population structure analysis, and genetic diversity assessment worldwide. However, their application to olive germplasm cultivated in the North-Western Himalayan region of India remains largely unexplored.

Therefore, the present investigation, conducted during 2025–2026, aimed to provide a comprehensive morpho-pomological and molecular characterization of olive cultivars grown under temperate Himalayan conditions of Jammu and Kashmir, India. Specifically, the objectives were to quantify phenotypic variability among cultivars using standardized vegetative, fruit, and stone descriptors; identify key traits contributing to cultivar differentiation through correlation and multivariate analyses; assess genetic diversity and cultivar relationships using SSR markers; and integrate morphological and molecular datasets to improve cultivar discrimination and germplasm characterization. To the best of our knowledge, this study represents the first integrated morpho-molecular assessment of olive cultivars cultivated in the North-Western Himalayan region of Jammu and Kashmir, providing valuable insights for cultivar authentication, genetic resource management, breeding programs, and the sustainable expansion of olive cultivation in emerging Himalayan production systems.

## 2 Materials and methods

### 2.1 Plant material

Morphological and pomological diversity of thirteen olive accessions, comprising eleven cultivars and two local selections, was evaluated during 2025–2026 at the Experimental Farm of the Division of Fruit Science, Sher-e-Kashmir University of Agricultural Sciences and Technology of Kashmir, Shalimar, Srinagar (34.1453° N, 74.8780° E; 1615 m above mean sea level).

The plant material consisted of seven-year-old, own-rooted trees of Moraiolo, Cornicabra Attica, Picholine, Nocellara Messinese, Zaituna, Itrana, Coratina, Carolea, Cerignola, Nocellara del Belice and Etnea, along with two locally maintained selections S1 and S2 (**Figures 1A, B**). Trees were uniform in vigor and maintained under standard orchard management practices, including irrigation, fertilization, pruning and plant protection, applied uniformly across all accessions. The experiment was conducted following a Randomized Complete Block Design (RCBD), with three biological replications for each of the thirteen olive accessions. Each tree constituted one biological replicate, resulting in a total of 39 experimental trees (13 accessions × 3 trees). Each replication included all thirteen accessions. Orchard management practices were maintained uniformly across all experimental trees throughout the study period to minimize variation arising from differential management.

**Figure 1:**
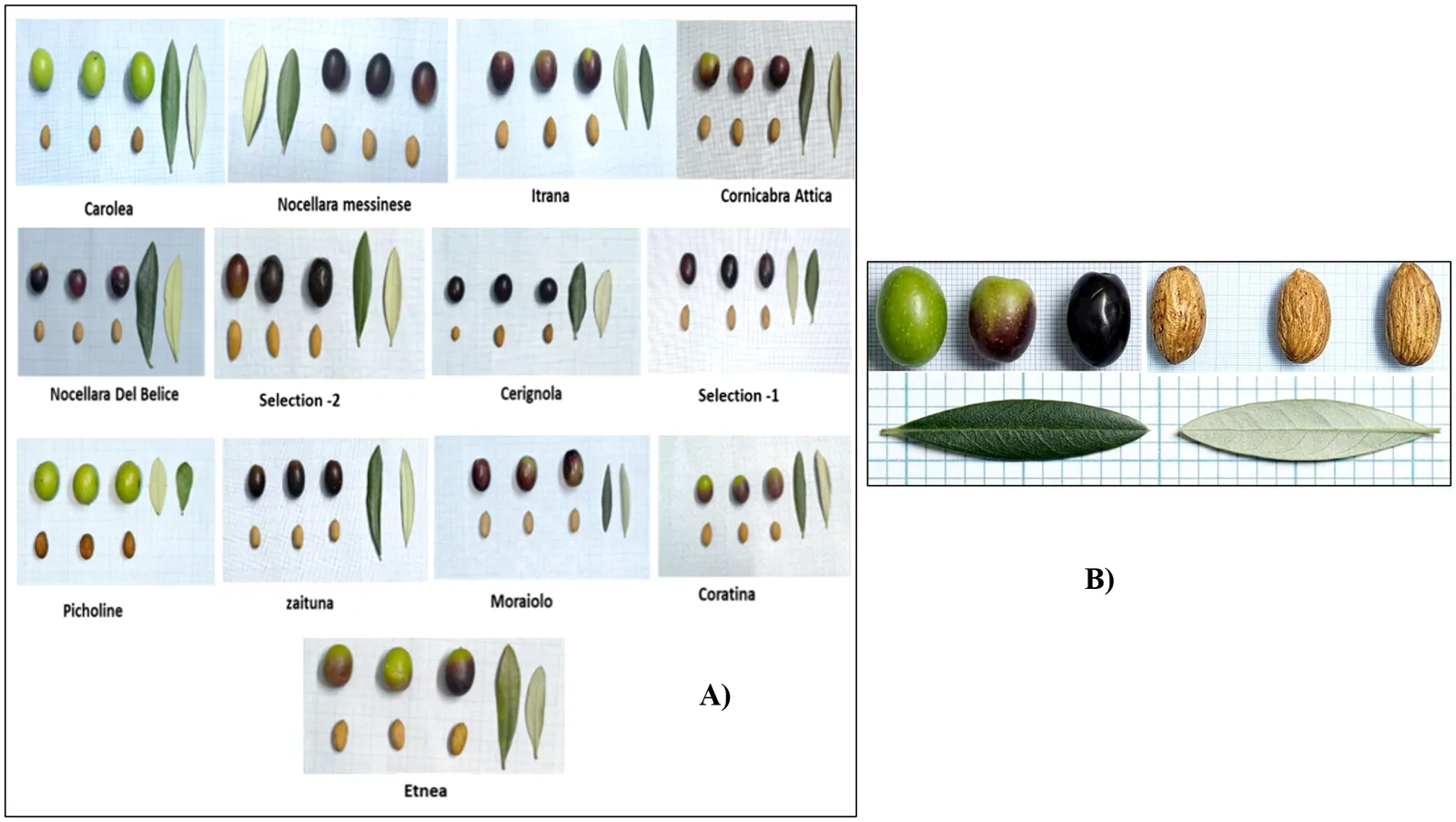
Representative morphological and pomological characteristics of thirteen olive cultivars. **(A)** Representative fruit, stone, and leaf morphology illustrating variation in qualitative morpho-pomological traits among the studied cultivars. **(B)** Close-up views showing variation in fruit shape, fruit color, stone morphology, and leaf architecture.

### 2.2 Trait measurement and characterization

A set of twenty-four morphological and pomological traits related to fruit, stone, and leaf characteristics was used to evaluate phenotypic diversity among thirteen olive accessions, with three biological replicate trees per accession, resulting in a total of 39 trees (**Tables 1**, **2**). For each accession, 30 mature fruits and 20 fully expanded leaves were randomly sampled from each of the three biological replicate trees, covering all canopy orientations (north, south, east, west, and center). Thus, a total of 1,170 fruits (13 accessions × 3 trees × 30 fruits) and 780 leaves (13 accessions × 3 trees × 20 leaves) were sampled. The fruits and leaves collected from each tree were treated as within-tree subsamples rather than independent biological replicates. For quantitative traits, measurements obtained from these subsamples were averaged within each tree to generate a single tree-level mean, which was used as the experimental observation in subsequent statistical analyses. Accordingly, the three trees per accession constituted the biological replicates, while the individual fruits and leaves served as within-tree subsamples.

**Table 1:** Frequency distribution of qualitative morphological traits among thirteen olive cultivars.

Characteristics	Category 1	Category 2	Category 3	Category 4
Fruit Shape (FS)	Circular (4)	Ovate (3)	Obovate (3)	Oblong (2), Elliptic (1)
Fruit Apex (FA)	Rounded (8)	Acute (5)	–	–
Fruit Base (FB)	Rounded (5)	Intermediate (5)	Truncate (2)	Rounded/Truncate mixed
MTD Position	Centered (6)	Toward Base (3)	Toward Apex (3)	–
Lenticel Density (LD)	Sparse (9)	Abundant (4)	–	–
Fruit Color (FC)	Purple (6)	Black (4)	Green (3)	–
Fruit Symmetry (FS)	Symmetric (6)	Weakly Asymmetric (4)	Strongly Asymmetric (2)	Asymmetric (1)
Fruit Nipple (FN)	Absent (5)	Tenuous (5)	Obvious (3)	–
Stone Shape (SS)	Oblong (5)	Elliptic (3)	Obovate (2)	Ovate (2)
Stone Surface (SSuf)	Smooth (6)	Scabrous (7)	–	–
Stone Apex (SA)	Obtuse (7)	Acute (3)	Rounded (2)	Truncate (1)
Stone Symmetry (SSym)	Symmetric (8)	Asymmetric (5)	–	–
Leaf Shape (LS)	Lanceolate (5)	Elliptic-lanceolate (4)	Elliptic (4)	–
Leaf Curvature (LC)	Flat (8)	Epinastic (4)	Hyponastic (2)	–

**Table 2:** Analysis of variance summary for quantitative traits of thirteen olive cultivars, showing significance of replication and cultivar effects, coefficient of variation (CV %), and least significant difference (LSD) at P ≤ 0.05.

Trait	Replication	Cultivar	CV (%)	LSD (0.05)
Fruit length (FL)	ns	***	1.76	0.64
Fruit diameter (FD)	ns	***	2.61	0.73
Stalk diameter (SD)	**	***	1.81	0.03
Fruit weight (FW)	***	***	1.02	0.07
Flesh-to-stone ratio (FSR)	***	***	0.95	0.06
Stone weight (SW)	***	***	0.71	0.01
Stone diameter (StD)	***	***	0.73	0.11
Number of grooves (NG)	ns	***	8.71	1.29
Leaf length (LL)	***	***	1.63	1.58
Leaf width (LW)	ns	***	2.71	0.68

** denotes significance at P ≤ 0.01. *** denotes significance at P ≤ 0.001.

Fruit traits included fruit length (FL, mm), fruit diameter (FD, mm), fruit stalk diameter (FSD, mm), fruit weight (FW, g), fruit shape, fruit apex (FA), fruit base (FB), position of maximum transverse diameter (MTD), lenticel density (LD), fruit symmetry (FS), fruit nipple (FN), flesh-to-stone ratio (FSR), and fruit color characteristics expressed as L*, a*, and b*. Quantitative fruit dimensions were measured instrumentally, whereas qualitative fruit descriptors were assessed visually according to standardized olive descriptor guidelines (UPOV, 2011; PPV&FRA, 2023). Fruit length, fruit diameter, and fruit stalk diameter were measured using a digital ABSOLUTE Digimatic caliper (Mitutoyo, Model 500-196-30; measuring range 0–150 mm, resolution 0.01 mm, accuracy ±0.02 mm), while fruit weight was determined using a digital balance with a readability of 0.01 g. Fruit color was first assessed visually using the standardized descriptor categories. For quantitative color characterization, measurements were recorded at the equatorial region of mature fruits using a portable colorimeter. In addition, digital images of representative fruits were captured under standardized illumination conditions and analyzed using *Color Analyzer–Color Lookup* (kamusoft), which provides color measurements in the CIELAB color space.

Stone traits included stone shape, stone surface, stone apex, stone symmetry, stone weight, stone diameter, and number of grooves. Stone shape, surface, apex, and symmetry were assessed visually according to the standardized descriptor guidelines, whereas stone weight and diameter were measured quantitatively. Stone weight was measured using the digital balance described above, and stone diameter was measured using the Mitutoyo digital caliper. The number of grooves was recorded according to the standardized descriptor assessment procedure.

Leaf traits included leaf length, leaf width, leaf shape, and longitudinal curvature. Leaf length and width were measured quantitatively using the same digital caliper, whereas leaf shape and longitudinal curvature were assessed visually according to the standardized descriptor guidelines (UPOV, 2011; PPV&FRA, 2023).

Measurements were obtained from individual trees, and the observations for each biological replicate tree were subsequently summarized as tree-level means for statistical analysis. Fruit images used for digital color analysis were captured under uniform and standardized illumination conditions to minimize variation attributable to image-acquisition conditions.

The flesh-to-stone ratio was calculated as:


$$\text{Flesh-to-stone ratio}=\frac{\text{Fruit weight}-\text{Stone weight}}{\text{Stone weight}}$$

### 2.3 Molecular characterization

Genomic DNA was extracted from young leaves of thirteen olive accessions following the cetyltrimethylammonium bromide (CTAB) method of Doyle and Doyle (1987) with minor modifications. DNA concentration and purity were determined using a NanoDrop™ spectrophotometer, while DNA integrity was assessed by electrophoresis on 0.8% agarose gels. Samples exhibiting A260/A280 ratios between 1.7 and 1.9 and intact high-molecular-weight DNA were diluted to a working concentration of 50 ng μL^−1^ for PCR amplification. To complement morpho-pomological characterization, ten previously reported SSR markers associated with olive genetic diversity were initially screened for polymorphism. Of these, three markers (UDO99-019, EMO3, and EMO88) produced polymorphic and reproducible amplification profiles and were selected for subsequent analysis, whereas the remaining seven monomorphic markers were excluded.

PCR amplification was performed in a 10 μL reaction volume containing genomic DNA, Taq DNA polymerase, reaction buffer, MgCl_2_, dNTPs, and primer pairs. Amplification was carried out in a thermal cycler with an initial denaturation at 94 °C for 5 min, followed by 35 cycles of denaturation at 94 °C for 45 s, primer-specific annealing for 45 s, extension at 72 °C for 1 min, and a final extension at 72 °C for 10 min.

Amplified products were resolved on 3% agarose gels using a 100 bp DNA ladder and visualized under UV illumination with a gel documentation system. Fragment sizes were estimated by comparison with the DNA ladder, and amplified fragments were assigned to allele classes based on their molecular size. The number of alleles (Na), observed heterozygosity (Ho), expected heterozygosity (He), polymorphism information content (PIC), Shannon’s diversity index (I), and effective number of alleles (Ne) were calculated using PowerMarker version 3.25.

### 2.4 Statistical analysis

Data were analyzed using a randomized complete block design, with cultivar as the treatment factor and three trees per cultivar as biological replications. Each tree constituted one biological experimental unit, giving a total of 39 biological units (13 cultivars × 3 trees). Across the entire experiment, 1,170 mature fruits (13 cultivars × 3 trees × 30 fruits) and 780 fully expanded leaves (13 cultivars × 3 trees × 20 leaves) were sampled. The fruits and leaves collected from each tree were treated as within-tree subsamples rather than independent biological replicates. Measurements from these subsamples were averaged within each tree to obtain a single tree-level value for each quantitative trait. Thus, ANOVA was conducted using 39 tree-level observations, with three biological replicates per cultivar, thereby avoiding pseudoreplication.

Analysis of variance (ANOVA) was performed to assess differences among accessions, and mean comparisons were conducted at P ≤ 0.05 using Duncan’s Multiple Range Test (DMRT).

Pearson’s correlation coefficients were calculated using cultivar mean values (n = 13) to evaluate the relationships among morphological and pomological traits. Principal component analysis (PCA) was used to identify major sources of phenotypic variation, and a biplot based on the first two principal components, principal component 1 and principal component 2 (PC1 and PC2) was constructed to visualize cultivar–trait associations. Hierarchical cluster analysis using Ward’s method and Euclidean distance was performed to classify olive germplasm based on phenotypic similarity. A heatmap was generated to illustrate patterns of trait variation among different accessions. Multiple linear regression analysis was performed using individual tree observations (n = 39) to maximize statistical power and evaluate the contribution of morphological, pomological and color traits to variation in fruit weight and stone weight. All statistical analyses and graphical visualizations were performed using R software (version 4.6.0), utilizing packages including agricolae, ggplot2, factoextra, corrplot, ggcorrplot and pheatmap. Although SSR markers are codominant, the observed allele-size profiles were converted into an allele-presence/absence matrix for the specific purpose of similarity-based clustering. Each detected allele was treated as a separate binary character and scored as 1 (present) or 0 (absent) across the evaluated germplam. Jaccard’s similarity coefficient was then calculated from the resulting matrix, and the similarity matrix was subjected to UPGMA (Unweighted Pair Group Method with Arithmetic Mean) clustering using MVSP version 3.22. This approach was adopted to compare multilocus allele profiles and was previously applied in olive cultivar studies using SSR data (Koehmstedt et al., 2011). The resulting dendrogram was therefore interpreted as a similarity-based representation of the observed allele profiles, rather than as a formal estimate of population-level genetic distance or genetic structure. Importantly, conversion to binary data was restricted to the clustering analysis; the original codominant allele data were retained for estimation of genetic diversity parameters, including number of alleles (Na), observed heterozygosity (Ho), expected heterozygosity (He), polymorphism information content (PIC), Shannon’s diversity index (I), and effective number of alleles (Ne). These parameters were calculated using PowerMarker version 3.25 and further validated using GenAlEx version 6.5. Graphical visualization and complementary statistical analyses were performed using R version 4.6.0.

## 3 Results

### 3.1 Qualitative morphological characterization of olive accessions

Qualitative descriptors revealed substantial phenotypic variability among the studied olive germplasm (**Table 1**). Fruit shape exhibited considerable diversity, with circular fruits being the most prevalent (4 cultivars), followed by ovate and obovate forms (3 cultivars each), while oblong and elliptic shapes were comparatively less frequent. Fruit apex was predominantly rounded (8 cultivars), whereas the remaining cultivars displayed an acute apex. Similarly, fruit base varied among rounded, intermediate, and truncate forms, reflecting distinct cultivar-specific pomological characteristics.

Variation in external fruit attributes was also pronounced. Fruit color ranged from green to purple and black, with purple being the dominant class. Lenticel density was primarily sparse (9 cultivars), while a smaller proportion exhibited abundant lenticels. Fruit symmetry varied from symmetric to strongly asymmetric types, indicating structural diversity. Nipple expression at the stylar end was absent in five cultivars, tenuous in five, and clearly pronounced in three cultivars, demonstrating variability in fruit tip morphology.

Stone characteristics further highlighted substantial heterogeneity. Stone shape was distributed among oblong, elliptic, obovate, and ovate categories, whereas surface texture was nearly evenly divided between smooth and scabrous types. Stone apex showed variation across obtuse, acute, rounded, and truncate forms, with the majority of cultivars exhibiting symmetric stones.

Leaf morphology also differed notably among cultivars. Leaf shapes included lanceolate, elliptic-lanceolate, and elliptic forms, while leaf curvature was predominantly flat, with fewer instances of epinastic and hyponastic orientations. Overall, the wide variation observed across fruit, stone, and leaf descriptors underscores the effectiveness of qualitative traits for preliminary characterization and discrimination of olive cultivars.

### 3.2 Variability in quantitative pomological and vegetative traits

Analysis of variance revealed highly significant (P ≤ 0.001) cultivar effects for all ten quantitative traits, indicating the presence of substantial variability among the studied olive germplasm (**Table 2**). Replication effects were non-significant for fruit length, fruit diameter, number of grooves, and leaf width, but significant for the remaining traits. Coefficients of variation were low for most characters, ranging from 0.71% (stone weight) to 8.71% (number of grooves), reflecting acceptable experimental precision and consistency of measurements.

Descriptive statistics further demonstrated wide phenotypic variation across accessions (**Table 3**). Fruit length ranged from 16.80 to 27.02 mm, fruit diameter from 13.87 to 20.10 mm, and fruit weight from 2.13 to 7.95 g. Flesh-to-stone ratio varied between 2.16 and 6.06, while leaf length and leaf width ranged from 41.40 to 77.03 mm and 8.60 to 22.99 mm, respectively. Among the traits, Fruit weight exhibited the highest coefficient of variation (41.36%), followed by flesh-to-stone ratio (29.12%), leaf width (27.93%), and stone weight (27.71%), indicating relatively greater variability in these traits among the evaluated germplasm.

**Table 3:** Descriptive statistics of ten quantitative morphological and pomological traits evaluated among thirteen olive cultivars grown under Kashmir conditions, showing the minimum (Min), maximum (Max), mean, standard deviation (SD), and coefficient of variation (CV).

Trait	Unit	Min	Max	Mean	SD	CV (%)
Fruit length (FL)	mm	16.80	27.02	21.95	3.22	14.67
Fruit diameter (FD)	mm	13.87	20.10	16.42	2.10	12.77
Stalk diameter (SD)	mm	0.75	1.32	1.01	0.16	15.84
Fruit weight (FW)	g	2.13	7.95	4.28	1.77	41.36
Flesh-to-stone ratio (FSR)	Unit less	2.16	6.06	4.19	1.22	29.12
Stone weight (SW)	g	0.49	1.19	0.83	0.23	27.71
Stone diameter (StD)	mm	6.72	10.73	9.14	1.28	14.00
Number of grooves (NG)	count	5.67	11.33	8.69	1.87	21.52
Leaf length (LL)	mm	41.40	77.03	57.76	13.61	23.56
Leaf width (LW)	mm	8.60	22.99	14.93	4.17	27.93

### 3.3 Cultivar mean performance

Significant differences among accessions were observed for all measured traits (**Supplementary Table 1**). The longest fruits were recorded in Picholine (27.02 mm), followed by S1 and S2, whereas Cerignola and Cornicabra Attica exhibited the shortest fruits. Fruit diameter was highest in S2 (20.10 mm), which was statistically at par with Carolea (19.93 mm), while the lowest values were observed in Nocellara Messinese.

Fruit weight was highest in S1 (7.95 g), followed by Carolea (6.48 g) and S2 (6.23 g), whereas Cerignola and Nocellara Messinese recorded the lowest values. The highest flesh-to-stone ratio was observed in S1 (6.06), followed by Carolea and S2. Stone weight was highest in Picholine (1.19 g), while maximum stone diameter was recorded in S1 and Itrana.

Variation was also evident in stone and leaf traits. Zaituna, S1, and S2 exhibited the highest number of grooves, whereas Moraiolo recorded the lowest. Leaf length was greatest in Nocellara del Belice, Coratina, and Zaituna, while comparatively shorter leaves were observed in S1, Moraiolo, and Etnea. Leaf width was highest in S2, followed by Nocellara del Belice and Cornicabra Attica, whereas Itrana had the narrowest leaves.

### 3.4 Quantitative fruit color characterization (CIELAB analysis)

Quantitative fruit color assessment using the CIELAB system revealed considerable variation among the thirteen olive accessions (**Supplementary Table 3**). The L* values ranged from 8.80 in Moraiolo to 63.53 in Carolea, indicating substantial differences in fruit surface lightness. The a* values ranged from −17.90 in Carolea to 17.23 in Cornicabra Attica, demonstrating marked variation along the green–red color axis. The b* values varied from −3.93 in Itrana to 46.03 in Picholine, indicating differences along the blue–yellow color axis.

Carolea and Picholine exhibited the highest L* values (63.53 and 62.53, respectively), whereas Moraiolo and Zaituna recorded the lowest values (8.80 and 8.87, respectively). The most negative a* value was recorded in Carolea (−17.90), while Cornicabra Attica showed the highest positive a* value (17.23). For b*, Picholine recorded the highest value (46.03), whereas Itrana exhibited the lowest (−3.93). Overall, the CIELAB coordinates demonstrated pronounced quantitative variation in fruit color among the evaluated olive germplasm.

### 3.5 Principal component analysis

Principal component analysis (PCA) summarized the multivariate structure of the quantitative trait dataset, with the first three principal components having eigenvalues greater than 1 and collectively explaining 80.4% of the total variance (**Table 4**, **Figure 2**). PC1 explained 48.3% of the variance, followed by PC2 (20.0%) and PC3 (12.1%). PC1 and PC2 together accounted for 68.3% of the total variance and were used for the primary two-dimensional visualization of accession distribution and trait loadings. Although PC3 met the eigenvalue >1 criterion and was therefore retained, it was not included in the PC1–PC2 biplot because the graphical interpretation was restricted to the first two components. PC4 had an eigenvalue of 0.89 and was therefore not considered a retained component under the Kaiser criterion. Fruit diameter, fruit weight, fruit length, stalk diameter, and stone weight showed relatively high positive loadings on PC1, indicating that these traits contributed substantially to variation represented along this axis. In contrast, leaf width, leaf length, and number of grooves showed stronger associations with PC2, contributing to the separation of cultivars along the second principal component. The variable correlation circle illustrated the associations of the quantitative traits with PC1 and PC2 (**Figure 3**). Trait contribution analysis further identified fruit diameter, fruit weight, stone weight, fruit length, and stalk diameter among the major contributors to the variation captured by the principal components.

**Table 4:** Eigenvalues, proportion of variance, and cumulative variance explained by principal components derived from ten quantitative morphological and pomological traits of thirteen olive cultivars.

Principal component	Eigenvalue	Variance explained (%)	Cumulative variance (%)
PC1	4.83	48.3	48.3
PC2	2.00	20.0	68.3
PC3	1.21	12.1	80.4
PC4	0.89	8.9	89.3
PC5	0.46	4.6	93.9
PC6	0.35	3.5	97.4
PC7	0.13	1.3	98.7
PC8	0.10	1.0	99.7
PC9	0.03	0.3	100.0
PC10	0.01	0.1	100.0

**Figure 2:**
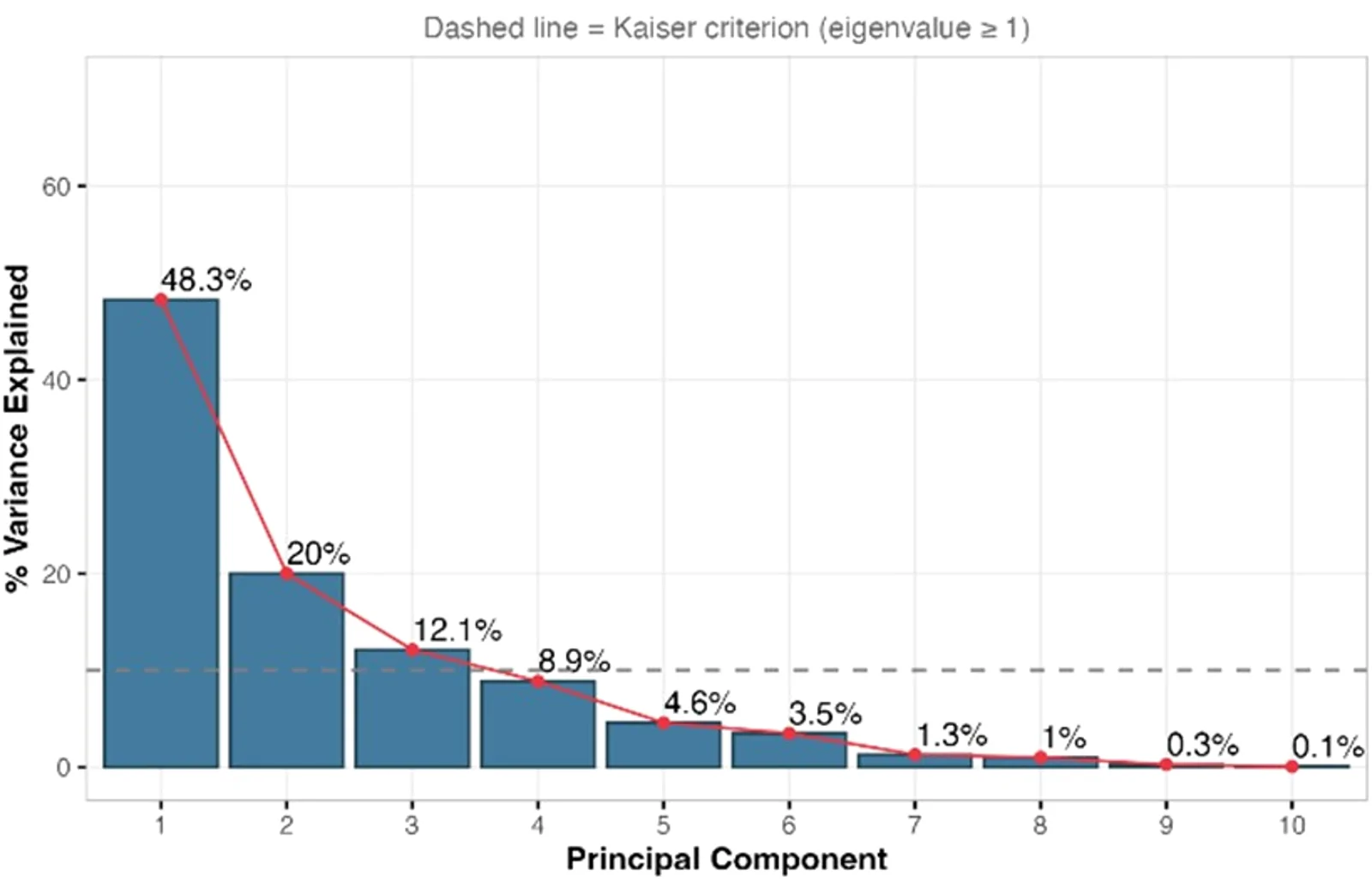
Scree plot showing the percentage of total phenotypic variation explained by successive principal components derived from morpho-pomological traits of thirteen olive cultivars.

**Figure 3:**
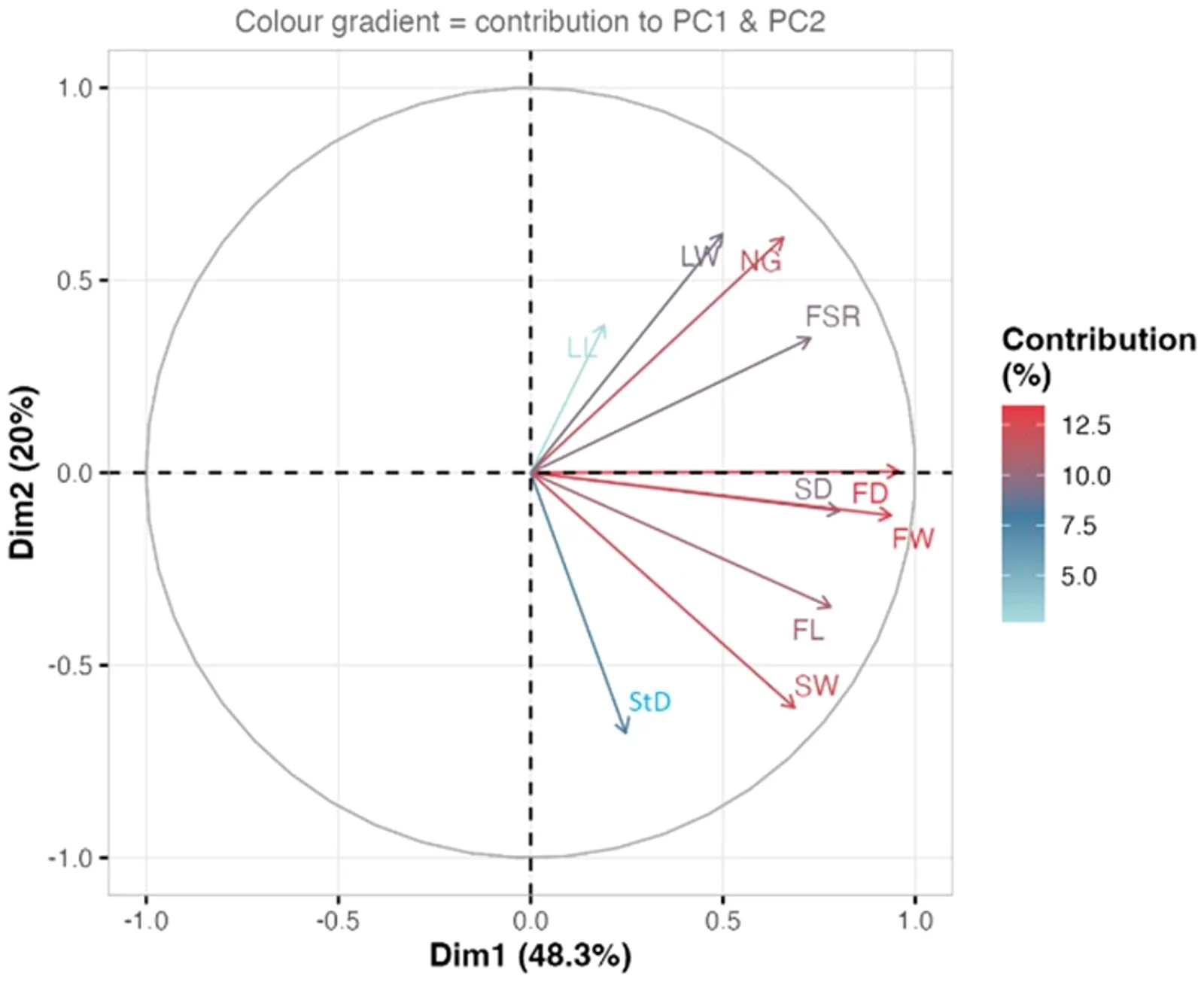
Variable correlation circle illustrating the relationships and contributions of morpho-pomological traits to the first two principal components (PC1 and PC2) in thirteen olive cultivars. FL, fruit length (mm); FD, fruit diameter (mm); StkD, stalk diameter (mm); FW, fruit weight (g); FSR, flesh-to-stone ratio; SW, stone weight (g); StD, stone diameter (mm); NG, number of grooves; LL, leaf length (mm); LW, leaf width (mm).

### 3.6 Distribution of cultivars in multivariate space

The PCA score plot demonstrated clear separation among cultivars based on morpho-pomological traits (**Figure 4A**). S1, S2, Carolea, and Picholine were positioned away from the central cluster, indicating distinct phenotypic characteristics and greater divergence from the remaining cultivars.

**Figure 4:**
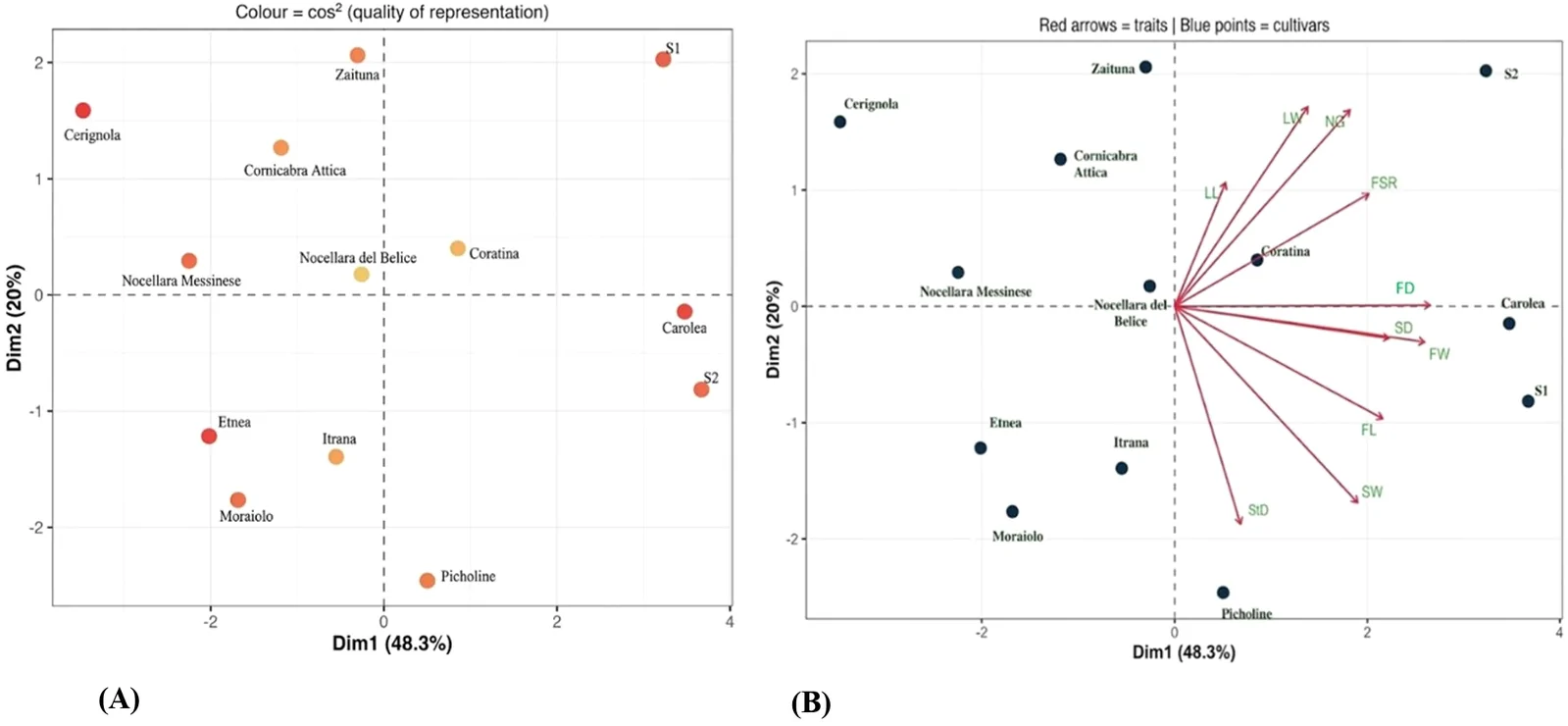
Principal component analysis (PCA) of thirteen olive cultivars based on morpho-pomological traits. **(A)** PCA score plot showing the distribution and separation of cultivars along the first two principal components (PC1 and PC2). **(B)** PCA biplot illustrating the relationships between cultivars and quantitative morpho-pomological traits, with cos² values representing the quality of representation of the traits on the PC1–PC2 axes. FL, fruit length (mm); FD, fruit diameter (mm); SD, stalk diameter (mm); FW, fruit weight (g); FSR, flesh-to-stone ratio; SW, stone weight (g); StD, stone diameter (mm); NG, number of grooves; LL, leaf length (mm); LW, leaf width (mm).

The PCA biplot further illustrated the association between cultivars and major contributing traits (**Figure 4B**). S2 was positioned in the direction of fruit diameter and leaf width, whereas S1 was more closely associated with fruit weight and flesh-to-stone ratio. Carolea and Picholine were positioned closer to fruit size-related vectors, indicating relatively distinct multivariate phenotypic profiles characterized by these traits. These spatial associations reflect the contribution of the respective traits to cultivar positioning within the PCA space and should not be interpreted as evidence of direct biological relationships or causal effects.

### 3.7 Hierarchical cluster analysis

Hierarchical clustering using Ward’s method grouped the cultivars into four major clusters based on overall similarity in fruit, stone, and leaf traits (**Figure 5**). Some cultivars formed closely related groups, whereas S1 and S2 appeared more distinct. The clustering pattern was consistent with PCA results (**Figures 4A, B**).

**Figure 5:**
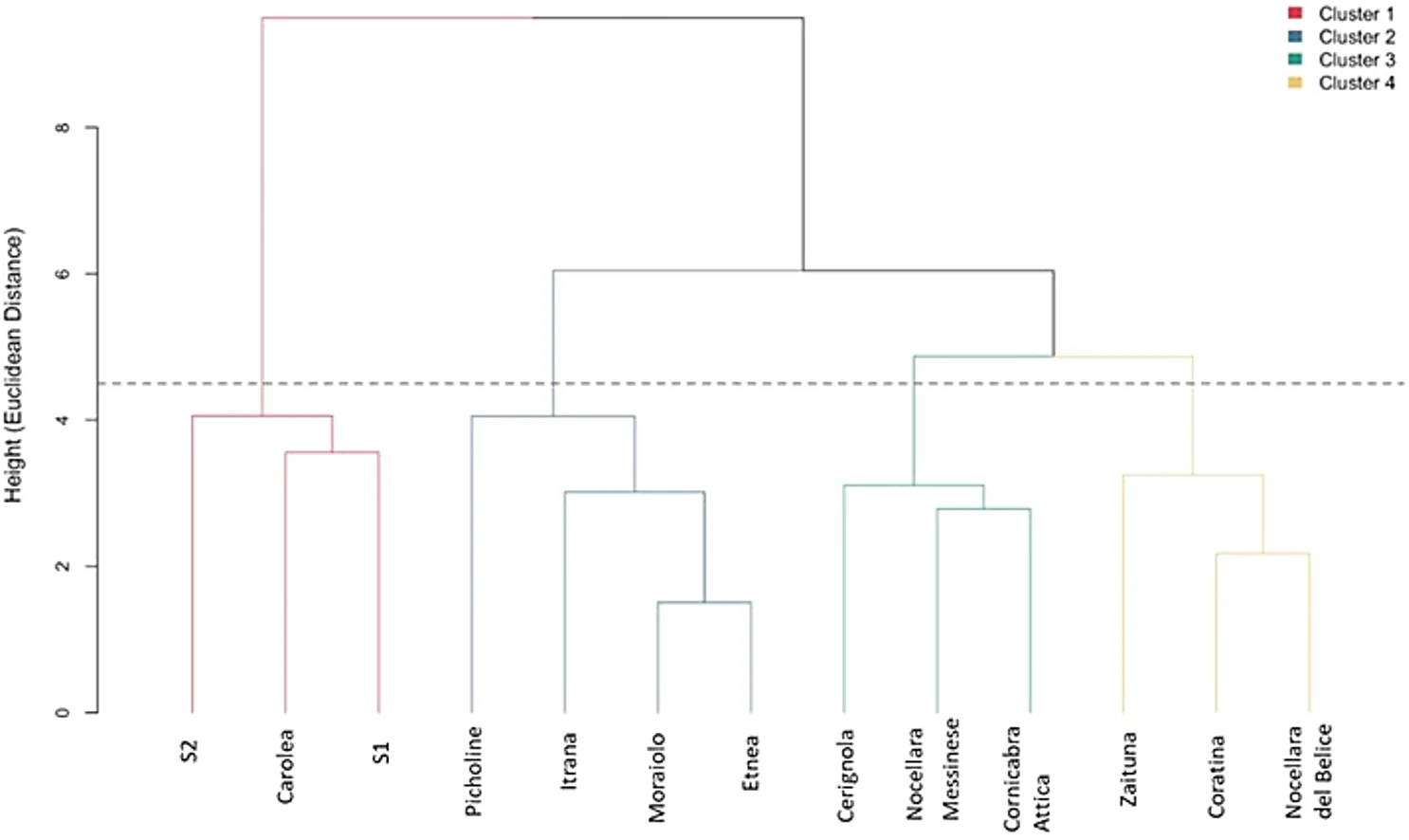
Hierarchical clustering dendrogram showing phenotypic relationships among thirteen olive cultivars based on morpho-pomological traits using Ward’s clustering method and Euclidean distance.

### 3.8 Correlation among traits

Pearson’s correlation analysis revealed significant associations among the studied morphological and pomological traits (**Supplementary Table 2**; **Figure 6**). Fruit weight showed strong positive correlations with fruit diameter (r = 0.859), fruit length (r = 0.716), stalk diameter (r = 0.664), flesh-to-stone ratio (r = 0.764), and stone weight (r = 0.699). Fruit length was also strongly correlated with stone weight (r = 0.847), while fruit diameter exhibited a high positive correlation with stalk diameter (r = 0.820).

**Figure 6:**
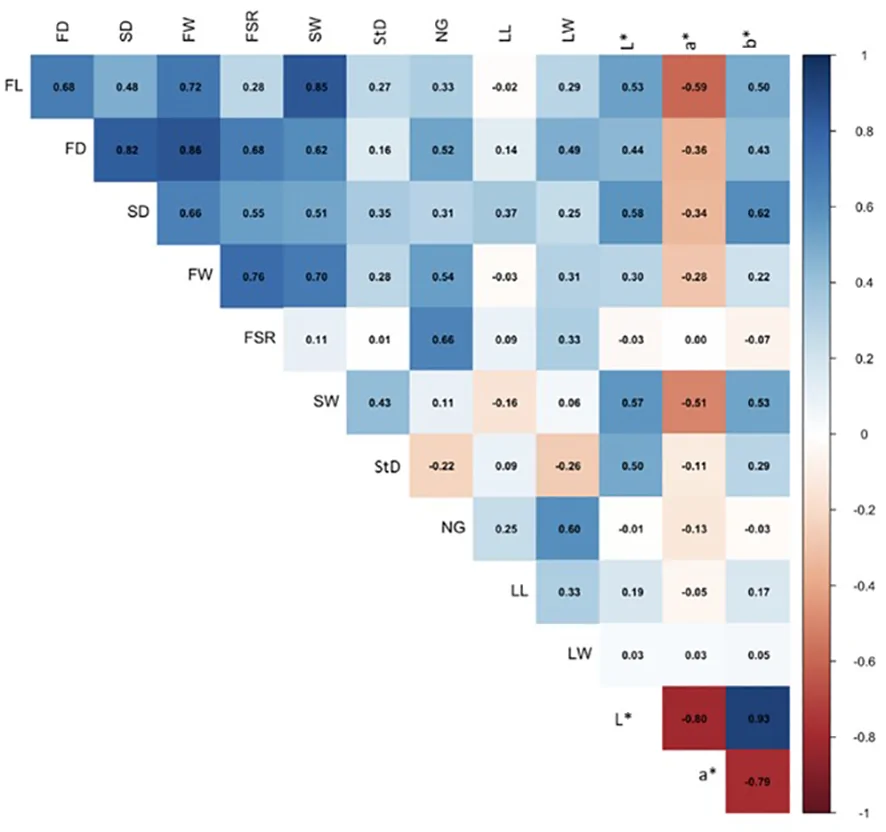
Pearson correlation heatmap showing relationships among quantitative morpho-pomological and fruit color traits of thirteen olive cultivars. Blue indicates positive correlations and red indicates negative correlations, with color intensity representing correlation strength. L* = lightness; a* = green–red coordinate; b* = blue–yellow coordinate.

Flesh-to-stone ratio showed significant positive associations with fruit diameter (r = 0.684) and number of grooves (r = 0.657). Leaf width was moderately correlated with number of grooves (r = 0.601), whereas leaf length exhibited no significant correlations with the other traits. Negative correlations were few and of low magnitude, with the highest observed between stalk diameter and leaf width (r = −0.264).

Color parameters showed distinct relationships, with lightness (L*) positively correlated with yellowness (b*) (r = 0.93) and negatively correlated with redness (a*) (r = −0.80).

### 3.9 Multiple regression analysis

Multiple regression analysis was performed to identify the traits contributing to variation in fruit weight and stone weight among olive germplasm (**Table 5**). The fruit weight model was highly significant (Coefficient of determination (R²) = 0.925, Adjusted R² = 0.895, P< 0.001), explaining 92.5% of the total variation. Fruit length emerged as a significant positive predictor of fruit weight (β = 0.162, P = 0.009), indicating that larger fruits tended to possess greater mass. Flesh-to-stone ratio also exhibited a significant positive association with fruit weight (β = 0.329, P = 0.047), suggesting that increased mesocarp development contributed substantially to overall fruit size. In contrast, leaf length showed a significant negative effect on fruit weight (β = −0.021, P = 0.030).

**Table 5:** Multiple linear regression models describing the effects of morphological, pomological and color traits on fruit weight and stone weight in olive cultivars.

Dependent trait	Independent trait	β (Regression coefficient)	t-value	p-value
Fruit Weight (FW, g)	Intercept	-9.178	-6.615	<0.001***
	Fruit Length (FL, mm)	0.162	2.805	0.009**
	Fruit Diameter (FD, mm)	0.224	1.530	0.138
	Stalk Diameter (SD, mm)	3.859	1.608	0.119
	Flesh: Stone Ratio (FSR)	0.329	2.079	0.047*
	Stone Diameter (StD, mm)	0.218	1.344	0.190
	Number of Grooves (NG)	0.069	0.818	0.420
	Leaf Length (LL, mm)	-0.021	-2.286	0.030*
	Leaf Width (LW, mm)	0.004	0.102	0.919
	L* (Lightness)	0.002	0.073	0.942
	a* (Redness)	-0.033	-1.057	0.300
	b* (Yellowness)	-0.043	-1.827	0.079.
Stone Weight (SW, g)	Intercept	-0.870	-4.138	<0.001***
	Fruit Length (FL, mm)	0.045	5.096	<0.001***
	Fruit Diameter (FD, mm)	0.039	1.776	0.087.
	Stalk Diameter (SD, mm)	0.557	1.532	0.137
	Flesh: Stone Ratio (FSR)	-0.098	-4.071	<0.001***
	Stone Diameter (StD, mm)	0.011	0.431	0.670
	Number of Grooves (NG)	0.020	1.533	0.137
	Leaf Length (LL, mm)	-0.004	-2.500	0.019*
	Leaf Width (LW, mm)	-0.012	-1.823	0.079.
	L* (Lightness)	0.002	0.565	0.577
	a* (Redness)	0.002	0.453	0.654
	b* (Yellowness)	-0.004	-1.029	0.312

Fruit weight model: R² = 0.925, Adjusted R² = 0.895, F = 30.47, P< 0.001; Stone weight model: R² = 0.897, Adjusted R² = 0.855, F = 21.30, P< 0.001. Significance levels: *P ≤ 0.05, **P ≤ 0.01, ***P ≤ 0.001,.P ≤ 0.10. [β, unstandardized regression coefficient; t, t-statistic; P, probability value. R², coefficient of determination].

The model for stone weight was likewise highly significant (R² = 0.897, Adjusted R² = 0.855, P< 0.001), accounting for 89.7% of the observed variation. Fruit length was the strongest positive predictor of stone weight (β = 0.045, P< 0.001), demonstrating a close association between fruit enlargement and stone development. Conversely, flesh-to-stone ratio exhibited a strong negative relationship with stone weight (β = −0.098, P< 0.001), indicating that cultivars with proportionally greater flesh tended to possess relatively lighter stones. Leaf length also exerted a significant negative influence on stone weight (β = −0.004, P = 0.019). Collectively, these results highlight fruit length and flesh-to-stone ratio as important variables associated with variation in fruit and stone weight in the evaluated olive germplasm (**Table 5**).

### 3.10 Molecular characterization and genetic diversity analysis

High-quality genomic DNA was successfully isolated from all thirteen olive accessions, yielding distinct high-molecular-weight DNA bands with minimal degradation, thereby confirming its suitability for SSR analysis (**Figure 7**). Among the ten SSR markers screened, three markers (UDO99-019, EMO3, and EMO88) were polymorphic and produced clear, reproducible, and easily scorable amplification profiles across the germplasm, whereas the remaining seven markers were monomorphic and therefore excluded from further analysis. The amplified fragments exhibited variation in allele sizes among accessions, indicating the presence of allelic polymorphism at the investigated loci (**Figure 8**, **Table 6**). Allele sizes were scored in base pairs and used to estimate codominant genetic diversity parameters. The three informative SSR markers generated a total of 11 alleles, with the number of alleles per locus ranging from 3 to 4 and an average of 3.67 alleles per locus (**Table 6**). The effective number of alleles (Ne) varied from 0.69 to 1.74, with a mean value of 1.35. Observed heterozygosity (Ho) ranged from 0.31 to 0.77, averaging 0.59, while expected heterozygosity (He) ranged from 0.32 to 0.42, with a mean value of 0.36. The polymorphism information content (PIC) values ranged from 0.31 to 0.43, with an average of 0.36, indicating a moderate level of marker informativeness. Similarly, Shannon’s diversity index (I) varied from 0.48 to 0.62, with an overall mean of 0.53. Among the SSR markers, UDO99–019 exhibited the highest marker informativeness, recording the greatest PIC value (0.43), Shannon’s diversity index (0.62), expected heterozygosity (0.42), and effective number of alleles (1.74), demonstrating its superior discriminatory ability for differentiating olive germplasm. In contrast, EMO88 displayed the lowest PIC (0.31) and expected heterozygosity (0.32), indicating comparatively lower polymorphism. The relatively high observed heterozygosity detected for EMO3 (0.77) suggests substantial heterozygosity among the evaluated accessions at this locus. Overall, the three polymorphic SSR loci revealed moderate marker-level diversity among the evaluated accessions (**Table 6**).

**Figure 7:**
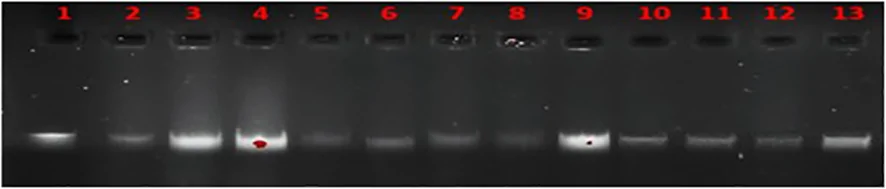
Agarose gel electrophoresis showing high-quality genomic DNA isolated from thirteen olive cultivars using the CTAB method. High-molecular-weight DNA bands with minimal smearing indicate DNA suitable for SSR analysis.

**Figure 8:**
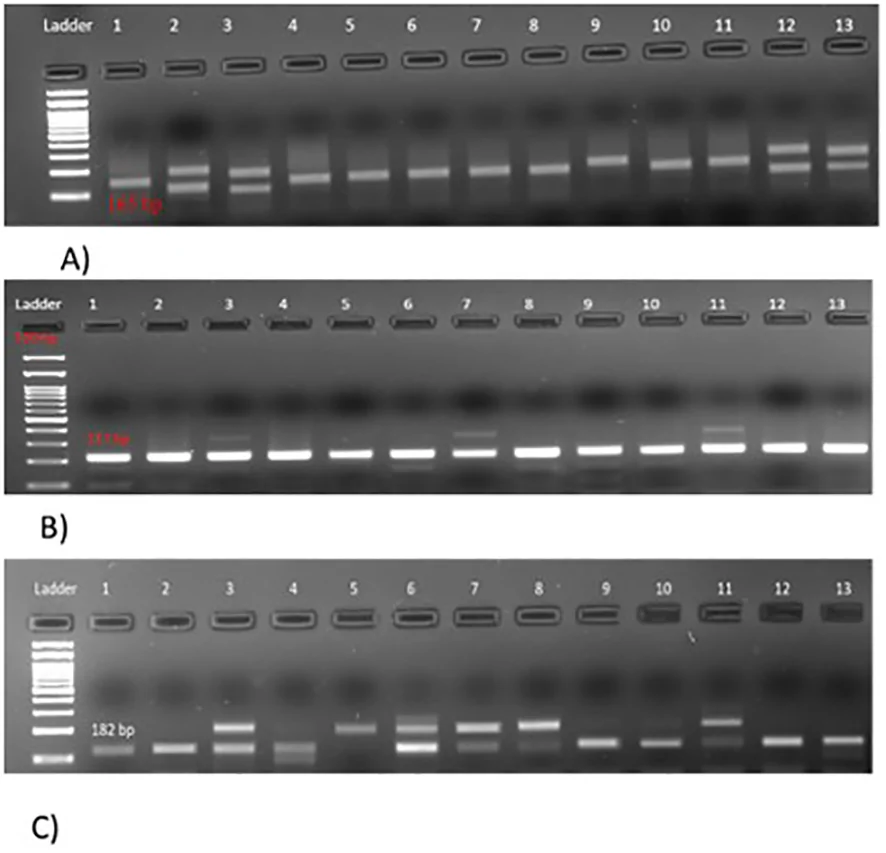
SSR amplification profiles of thirteen olive cultivars generated using polymorphic markers: **(A)** UDO99-019, **(B)** EMO3, and **(C)** EMO88. Differences in banding patterns among cultivars indicate allelic polymorphism suitable for cultivar discrimination.

**Table 6:** Characteristics and genetic diversity statistics of the polymorphic SSR markers used for molecular characterization of thirteen olive cultivars.

Marker	Forward primer	Reverse primer	Annealing temperature (°C)	Size range (bp)	PIC	I	Na	Ne	Ho	He	Reference
UDO99-019	TCCCTTGTAGCCTCGTCTTG	GGCCTGATCATCGATACCTC	57	125-165	0.43	0.62	3	1.74	0.31	0.42	(Cipriani et al., 2002); (Koehmstedt et al., 2011)
EMO3	GGTGTAGCCCAAGCCCTTAT	TGCATGACCGTGGTGTAAGT	60	94–217	0.34	0.48	4	1.63	0.77	0.35	(Yadav et al., 2021); (Koehmstedt et al., 2011)
EMO88	TGACCATCTCAAAACCATTCA	TTGCCCGGCTGTAGTTGTAT	46	178–198	0.31	0.48	4	0.69	0.69	0.32	(Koehmstedt et al., 2011)
Overall Mean					0.36	0.53	3.67	1.35	0.59	0.36	

PIC, polymorphic information content; I, Shannon’s diversity index; Ne, effective number of alleles; Ho, Observed Heterozygosity; He, Expected Heterozygosity. Values were calculated from binary presence/absence data generated from polymorphic SSR loci across thirteen olive cultivars. Ne was estimated as $1/\left(p^{2}+q^{2}\right)$.

For SSR-based relationship analysis, the allele data were subsequently transformed into a binary presence (1) and absence (0) matrix, and Jaccard’s similarity coefficient was calculated. The resulting similarity matrix was subjected to UPGMA clustering to infer genetic relationships among the thirteen olive accessions (**Figure 9**). The dendrogram resolved the accessions into three major clusters. Cluster I comprised Nocellara Messinese, Moraiolo, S1, and S2. Cluster II, the largest group, included Zaituna, Coratina, Carolea, Itrana, Cornicabra Attica, Cerignola, Nocellara del Belice, and Etnea. In contrast, Picholine formed a distinct Cluster III, indicating a comparatively distinct SSR allele profile relative to the remaining evaluated accessions. The clustering pattern revealed differences in SSR allele-profile similarity among the evaluated accessions under temperate Himalayan conditions.

**Figure 9:**
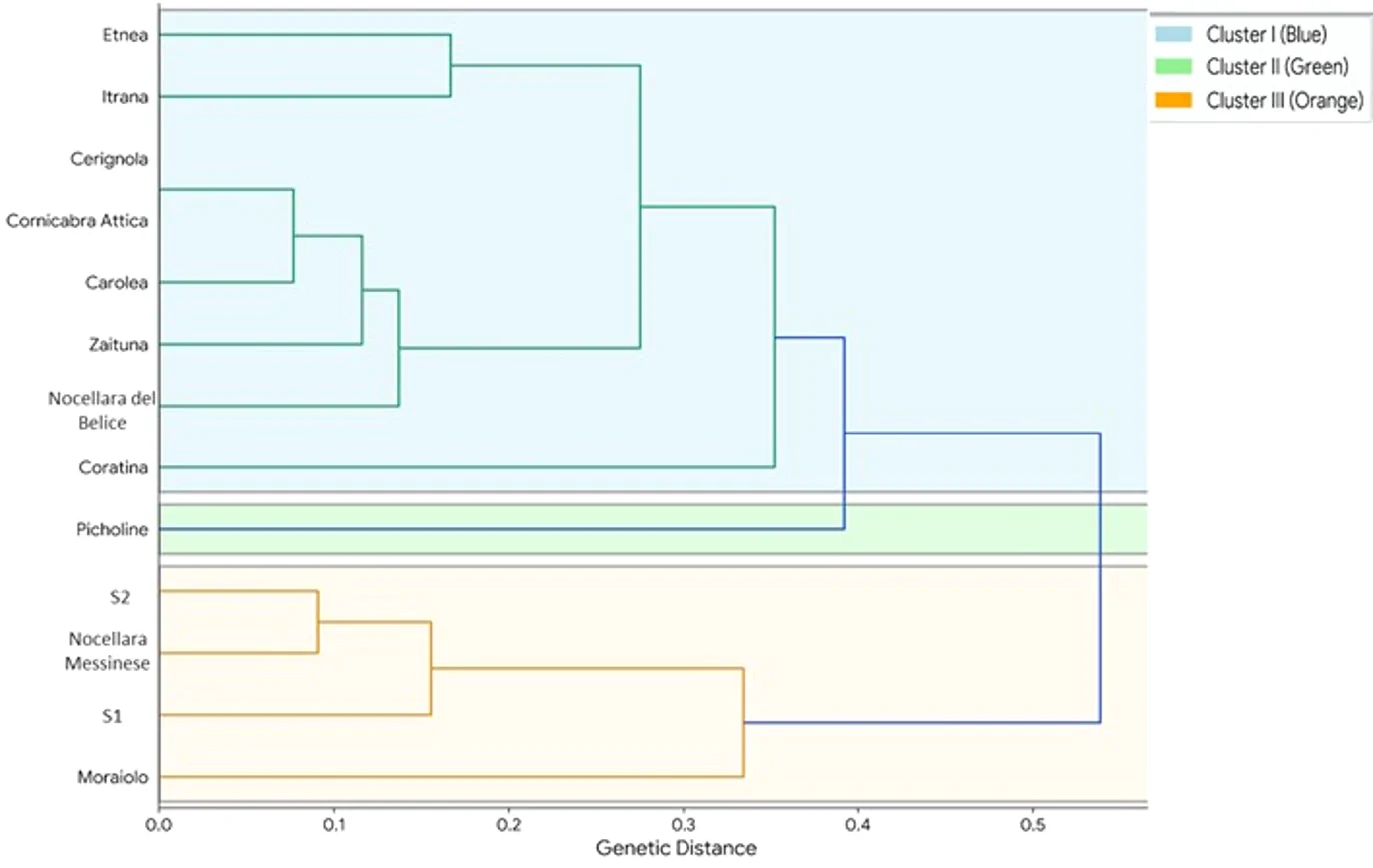
UPGMA dendrogram showing genetic relationships among thirteen olive cultivars based on polymorphic SSR markers (UDO99-019, EMO3, and EMO88). Clustering was performed using Jaccard similarity coefficients and the UPGMA algorithm, resulting in three major genetic clusters.

## 4 Discussion

The present study revealed substantial phenotypic differentiation among thirteen olive accessions evaluated under temperate Kashmir conditions. Significant variation across all ten quantitative traits, together with marked differences in qualitative fruit, stone and leaf descriptors, demonstrated the discriminatory value of the evaluated morpho-pomological traits under the prevailing experimental conditions. Such diversity is consistent with the broad phenotypic variability documented in olive, a clonally propagated perennial shaped by a long history of domestication, selection and cultivation across diverse agro-climatic environments. Previous investigations in Jammu and Kashmir have primarily emphasized cultivar performance, yield attributes, oil characteristics and basic variability parameters (Kumar et al., 2020, 2021). The present study extends this regional work by integrating standardized morpho-pomological characterization with correlation, multivariate and regression analyses and SSR-based molecular profiling. Because phenotypic evaluation was restricted to a single location and experimental period, however, the observed differences are interpreted as genotype-associated phenotypic differentiation under the prevailing conditions, rather than as direct evidence of genetic determination or specific environmental adaptation.

### 4.1 Phenotypic variability and cultivar differentiation under temperate Kashmir conditions

The magnitude and pattern of phenotypic variation observed were broadly consistent with diversity reported in olive germplasm from contrasting production environments. Khadivi et al. (2022) documented considerable variation in fruit, stone and leaf characteristics among 24 cultivars, particularly for fruit weight, fruit dimensions, fruit color and vegetative descriptors, whereas Dehghan-Seresht et al. (2025) reported significant differences among ten cultivars for fruit dimensions, weight and stone characteristics. Differences in trait magnitude among studies are nevertheless expected because phenotypic expression may vary with genotype composition, climate, soil, tree age, orchard management, developmental stage and measurement procedures. Thus, the significant cultivar differences observed in the present study reflect phenotypic variation expressed under the evaluated Kashmir conditions and should not be interpreted as evidence of heritable variation, greater genetic diversity, or environmental adaptation, particularly given that heritability and variance components were not estimated and only a limited number of polymorphic SSR markers were evaluated.

Qualitative descriptors provided additional discriminatory resolution. Differences in fruit shape, apex and base configuration, symmetry, color, lenticel density and nipple development, together with variation in stone shape, apex, surface characteristics, symmetry and groove pattern, are consistent with previous reports identifying fruit and endocarp traits as informative descriptors for olive cultivar characterization. Cantini et al. (1999) demonstrated their value for preliminary cultivar classification, while Ozkaya et al. (2006); Hannachi et al. (2008) and Zaher et al. (2011) emphasized the discriminatory contribution of fruit and stone characteristics. Lazović et al. (2018) likewise confirmed their usefulness for cultivar identification, and Titouh et al. (2021) identified fruit shape, fruit weight, stone shape, stone symmetry and pulp-related traits as important contributors to phenotypic grouping. The convergence of these findings suggests that fruit and stone descriptors capture biologically informative differences in developmental and structural attributes, particularly when evaluated in combination.

Morphological distinctness, however, should not be equated with genetic differentiation. Phenotypic traits represent the integrated expression of genotype and environment, and descriptors such as fruit color and lenticel characteristics may vary with maturity, temperature, irradiance and other environmental factors. Instrumental color studies have similarly demonstrated cultivar-associated differences alongside maturation-related changes in olive pigmentation (Rotondi et al., 2003; Ganino et al., 2006; D’Imperio et al., 2011). Accordingly, these qualitative traits are best considered phenotypically informative under the evaluated conditions rather than exclusively genetically determined. This limitation is particularly relevant for cultivar authentication: Rotondi et al. (2003) showed that although morphological and pomological traits could reflect cultivar relationships, molecular markers were required to resolve closely related cultivars and detect synonymies or mislabeling.

Fruit-related quantitative traits provided further phenotypic resolution. Fruit weight ranged from 2.13 to 7.95 g, while fruit length, diameter, stone weight and flesh-to-stone ratio also varied substantially. These traits represent different aspects of fruit and endocarp development, with flesh-to-stone ratio additionally reflecting the relative contribution of mesocarp to the non-edible endocarp. The comparatively high coefficients of variation for fruit weight (41.36%) and flesh-to-stone ratio (29.12%) indicate relatively greater variability in these traits within the evaluated material. Similar importance of fruit biometric and pulp-related traits has been reported by Khadivi et al. (2022); Dehghan-Seresht et al. (2025) and Titouh et al. (2021). Their variation in the present study is therefore relevant for phenotypic characterization and preliminary selection, although their stability and value as selection criteria require validation across environments.

Vegetative traits contributed additional information but were less prominent than fruit-related variables in the multivariate structure. Khadivi et al. (2022) reported substantial diversity in olive leaf morphology, indicating that the relative contribution of vegetative traits may differ among germplasm collections and environments. Thus, their comparatively lower contribution in the present dataset does not imply limited discriminatory value; rather, their inclusion alongside fruit and stone descriptors provides a more comprehensive representation of cultivar phenotype.

The practical relevance of morpho-pomological characterization is particularly important in olive because its perennial nature and prolonged juvenile phase can complicate early cultivar evaluation. Reliable descriptors can facilitate preliminary identification and characterization before long-term agronomic performance is established. Hosseini-Mazinani et al. (2004) emphasized the importance of reliable cultivar characterization, while Cantini et al. (1999); Ozkaya et al. (2006) and Zaher et al. (2011) demonstrated the practical utility of morphological descriptors for cultivar identification. Nevertheless, morphological and genomic classifications need not be congruent. Casilla García et al. (2025), for example, reported discrepancies between morphological and genomic groupings, reinforcing the potential for phenotypic similarity to mask underlying genetic differences.

Overall, the findings support the use of standardized morpho-pomological descriptors—particularly fruit dimensions, fruit weight, flesh-to-stone ratio and selected stone characteristics—as a first level of phenotypic discrimination among the evaluated genotypes. Their alignment with DUS characterization principles provides a reproducible framework for documenting phenotypic distinctness. However, the present results should be regarded as a baseline assessment under Kashmir conditions; multi-year and multi-location evaluation will be necessary to establish trait stability, genotype × environment effects and the broader agronomic value of the identified material.

### 4.2 Quantitative fruit color characterization (CIELAB analysis)

The considerable variation in L*, a*, and b* values among the evaluated germplasm indicates substantial phenotypic variation in fruit color under temperate Himalayan conditions. The wide range of L* values reflected differences in fruit surface lightness, while variation in a* and b* indicated differences along the green–red and blue–yellow color axes, respectively. Carolea and Picholine showed comparatively higher L* values, whereas Moraiolo and Zaituna exhibited lower values. The negative a* values in Carolea and Picholine indicated stronger green components, while positive values in cultivars such as Cornicabra Attica and Coratina reflected greater red components. Picholine recorded the highest b* value, indicating a stronger yellow component, whereas Itrana showed a negative b* value.

These findings are consistent with previous studies reporting considerable variation in instrumental fruit color among olive germplasm (Rotondi et al., 2003; D’Imperio et al., 2011; Khadivi et al., 2022; Dehghan-Seresht et al., 2025). The present results further demonstrate the usefulness of CIELAB coordinates as objective descriptors for complementing qualitative fruit-color assessment and improving olive cultivar characterization. However, these differences represent phenotypic variation expressed under the evaluated conditions and should not be interpreted as direct evidence of genetic determination, as fruit color may also be influenced by maturity stage and environmental conditions.

### 4.3 Trait relationships and fruit-size variation

Pearson’s correlation analysis revealed a predominantly positive association structure, with the strongest relationships concentrated among fruit size-related traits. Fruit weight was strongly associated with fruit diameter (*r* = 0.859), flesh-to-stone ratio (*r* = 0.764), fruit length (*r* = 0.716), stone weight (*r* = 0.699) and stalk diameter (*r* = 0.664), while fruit length was strongly associated with stone weight (*r* = 0.847) and fruit diameter with stalk diameter (*r* = 0.820). Comparable associations among fruit dimensions, weight and stone traits have been reported in olive (Khadivi et al., 2022; Dehghan-Seresht et al., 2025), suggesting that these descriptors commonly constitute an interconnected component of pomological variation. The association between fruit length and stone weight may reflect coordinated development of pericarp and endocarp tissues, although correlation alone cannot establish a developmental mechanism.

Flesh-to-stone ratio was positively associated with fruit weight (*r* = 0.764) and fruit diameter (*r* = 0.684), but only weakly with stone weight (*r* = 0.105), indicating that variation in fruit mass was more closely associated with mesocarp contribution than with proportional increases in stone mass within the evaluated material. This relationship is relevant to fruit characterization because greater mesocarp proportion may be advantageous for table-olive applications. However, the association should not be interpreted causally, particularly under a single environmental setting. The positive relationship with number of grooves (*r* = 0.657) also suggests covariance between some stone characteristics and fruit composition, although its biological basis remains unresolved.

Vegetative–reproductive associations were generally weaker. Leaf width showed moderate associations with number of grooves (*r* = 0.601) and fruit diameter (*r* = 0.488), whereas leaf length showed weak relationships with fruit and stone traits. This pattern indicates that fruit-size variation was more closely structured by fruit and stone characteristics than by leaf dimensions in the present material, although weak correlations do not imply biological independence and may vary with germplasm and environment (Khadivi et al., 2022).

Overall, fruit diameter, fruit length and fruit weight emerged as closely associated descriptors of fruit-size variation, while flesh-to-stone ratio provided complementary information on fruit composition. These relationships may have value for preliminary indirect selection and cultivar characterization, consistent with previous olive studies (Corrado et al., 2009; Khadivi et al., 2022). Importantly, pairwise correlations indicate association rather than causation, and their magnitude does not necessarily predict the independent contribution of a trait in regression models. The correlation structure therefore identifies coordinated phenotypic variation, whereas the developmental mechanisms underlying these associations require validation through targeted developmental and multi-environment studies.

### 4.4 Multivariate structure and key determinants of variability

PCA showed that phenotypic differentiation was driven predominantly by fruit- and stone-related characteristics, with PC1 capturing the major dimension associated with fruit size and related pomological attributes. This agrees with previous olive studies in which fruit and stone biometric traits contributed strongly to multivariate differentiation (Khadivi et al., 2022; Dehghan-Seresht et al., 2025). The recurrence of this pattern across geographically diverse collections supports the discriminatory value of these traits, although their relative contribution may vary with germplasm and growing conditions.

PC2 was more strongly associated with leaf traits and number of grooves, indicating an additional dimension of phenotypic variation beyond fruit size. PC3, which also met the eigenvalue >1 criterion, accounted for a further 12.1% of the total variance and therefore represented an additional informative axis of phenotypic variation. Because PCA components represent patterns of statistical covariance rather than discrete biological processes, they should be interpreted as dataset-specific axes of phenotypic variation rather than as independent developmental pathways or productivity mechanisms.

The separation of S1, S2, Carolea and Picholine in multivariate space reflects contrasting combinations of measured traits rather than genetic divergence or environmental adaptation. The association of S1 with fruit weight and flesh-to-stone ratio and S2 with fruit diameter and leaf width identifies distinct phenotypic profiles and supports their consideration as promising material for further evaluation, rather than evidence of inherent superiority.

The present PCA is broadly consistent with previous multivariate characterization of olive germplasm (Belaj et al., 2012; Dehghan-Seresht et al., 2025), although differences in individual trait contributions are expected because PCA depends on germplasm composition, descriptors, environmental conditions and covariance structure. The PCA therefore provides a dataset-specific assessment of phenotypic structure under Kashmir conditions, appropriately complemented by SSR analysis to determine whether phenotypic patterns correspond to molecular differentiation.

### 4.5 Cluster analysis and phenotypic divergence

Hierarchical Cluster Analysis (HCA) grouped the genotypes according to similarity in their overall multivariate phenotypic profiles, with S1 and S2 occupying distinct positions consistent with their contrasting fruit-related characteristics. Broad concordance between HCA and PCA strengthens the evidence for structure within the phenotypic dataset, consistent with the use of complementary multivariate approaches for olive cultivar discrimination (Khadivi et al., 2022; Dehghan-Seresht et al., 2025). Differences in grouping among studies are nevertheless expected because clustering is sensitive to germplasm composition, descriptors, environmental conditions and covariance structure. Because the present HCA was based exclusively on phenotypic measurements, cluster membership represents phenotypic similarity rather than direct genetic relatedness. This distinction is important in olive because environmental conditions, orchard management and phenotypic plasticity can modify morphological expression. Rotondi et al. (2003) demonstrated that morphological relationships may be insufficient for unequivocal cultivar authentication, while Casilla García et al. (2025) similarly reported discrepancies between morphological and genomic groupings. Thus, the present clusters should not be interpreted as evidence of genetic divergence, geographic differentiation, environmental adaptation or selection history.

The distinct positions of S1 and S2 identify contrasting phenotypic profiles that warrant further evaluation, particularly for fruit and stone characteristics. Likewise, contrasting clusters may provide useful phenotypic diversity for subsequent selection and breeding, but parental value should be evaluated using molecular relationships, reproductive compatibility and agronomic performance rather than cluster distance alone. HCA therefore complements PCA in resolving phenotypic structure, while SSR analysis provides independent evidence of the underlying molecular relationships.

### 4.6 Multiple regression analysis of fruit and stone weight

Multiple regression identified fruit length as a consistent positive predictor of both fruit and stone weight, reinforcing the importance of fruit dimensional traits in explaining pomological variation, consistent with previous olive germplasm studies (Khadivi et al., 2022). Fruit length remained significant for fruit weight (estimate = 0.162, *P* = 0.009) and stone weight (estimate = 0.045, *P* < 0.001). However, comparison with the correlation analysis showed that strong pairwise associations did not always translate into significant contributions in the multivariable models. Fruit diameter and stalk diameter, for instance, were significantly correlated with fruit weight (*r* = 0.859 and 0.664, respectively), but neither remained significant after simultaneous adjustment for the other predictors (*P* = 0.138 and 0.119). This likely reflects the substantial covariance among fruit dimensional traits, particularly the associations of fruit diameter with fruit length (*r* = 0.685) and stalk diameter (*r* = 0.820), resulting in overlapping explanatory information and a reduced unique contribution of individual traits in the regression model.

Conversely, leaf length exhibited weak and non-significant pairwise associations with fruit weight (*r* = −0.028) and stone weight (*r* = −0.155), yet showed significant negative coefficients in both regression models (*P* = 0.030 and 0.019, respectively). This indicates that its conditional association became apparent after the effects of the other predictors were considered simultaneously. Such differences are not contradictory but reflect the distinct information provided by the two approaches: Pearson correlation describes the overall pairwise association between traits, whereas multiple regression estimates the association of each predictor conditional on the remaining variables in the model. Thus, the regression analysis complements the correlation structure by distinguishing associations attributable to shared variation from those that remain detectable after adjustment for other measured traits.

The flesh-to-stone ratio showed a positive relationship with fruit weight in both analyses (*r* = 0.764; regression *P* = 0.047), whereas its association with stone weight differed markedly. Although its pairwise correlation with stone weight was weak and non-significant (*r* = 0.105), it exhibited a significant negative regression coefficient (estimate = −0.098, *P* < 0.001). This result warrants caution because the flesh-to-stone ratio is mathematically derived from fruit and stone components and is therefore not fully independent of the response variables. Its regression coefficient should consequently be interpreted as a statistical association rather than evidence of an independent biological effect. Similarly, the negative conditional association of leaf length with fruit and stone weight should not be interpreted as evidence that greater vegetative growth directly suppresses reproductive development, as relationships between vegetative and reproductive traits may vary with genotype and environmental conditions (Dehghan-Seresht et al., 2025).

The fitted models explained a substantial proportion of the observed variation in fruit weight (*R*² = 0.925; adjusted *R*² = 0.895) and stone weight (*R*² = 0.897; adjusted *R*² = 0.855). Nevertheless, given the 39 observations, multiple predictors and the covariance among several fruit traits, these models should be interpreted primarily as exploratory evidence of trait associations rather than as validated predictive or causal models. Overall, the regression findings complement the correlation and multivariate analyses by identifying fruit dimensional traits, particularly fruit length, as important variables associated with fruit and stone mass. The potential value of these traits for indirect selection and prediction should be confirmed using larger datasets and independent multi-environment evaluations.

### 4.7 SSR-based genetic diversity and relationship analysis

SSR-based genotyping provided an independent molecular perspective on genetic variation among the thirteen olive accessions and complemented the phenotypic and morpho-pomological characterization. The three polymorphic loci, UDO99-019, EMO3, and EMO88, collectively detected eleven alleles, corresponding to a mean of 3.67 alleles per locus. This confirms that the assayed loci were capable of detecting allelic variation among the evaluated germplasm despite the limited number of polymorphic markers retained for analysis. SSR markers are widely recognized for their reproducibility, codominant inheritance and discriminatory potential in olive, and have been successfully applied to cultivar identification, genetic diversity assessment and germplasm characterization across geographically diverse collections (Carriero et al., 2002; Cipriani et al., 2002; Li et al., 2020).

Marker informativeness varied among the three loci, with UDO99–019 exhibiting the highest PIC (0.43), Shannon’s diversity index (0.62) and effective number of alleles (1.74), indicating greater discriminatory potential within the present germplasm set. Such locus-specific variation is expected because SSR informativeness depends on both allele number and allele-frequency distribution. PIC is widely used to assess marker informativeness, with higher values indicating greater discriminatory capacity (Botstein et al., 1980). Similar variation in allelic diversity and marker performance has been reported among olive SSR loci (Koehmstedt et al., 2011; Yadav et al., 2021), supporting the utility of selected polymorphic loci for cultivar-level characterization.

The mean effective number of alleles (1.62), expected heterozygosity (He = 0.36) and Shannon’s diversity index (I = 0.53) indicate detectable marker-level variation within the evaluated germplasm. Observed heterozygosity (Ho = 0.59) exceeded expected heterozygosity, indicating relatively high heterozygosity at the assayed loci. High heterozygosity is commonly reported in olive and has been associated with its predominantly outcrossing reproductive biology, long cultivation history and extensive vegetative propagation (Sefc et al., 2000; Belaj et al., 2004; Koehmstedt et al., 2011). However, because these estimates were derived from only three polymorphic loci, they should be regarded as marker-specific rather than representative of genome-wide heterozygosity.

The level of allelic variation should likewise be interpreted in the context of the germplasm and markers examined. SSR diversity estimates can vary with sample size and composition, geographical origin, population structure and the particular loci employed (Sefc et al., 2000). Thus, the moderate marker-level diversity observed here should not be interpreted as evidence of limited genetic variation in olive germplasm generally, but rather as a characteristic of the thirteen accessions and three polymorphic loci evaluated (Koehmstedt et al., 2011).

The UPGMA analysis based on Jaccard’s similarity coefficients separated the thirteen accessions into three groups based on the allelic profiles detected by the assayed loci. Nocellara Messinese, Moraiolo, S1 and S2 showed relatively close molecular similarity, whereas Picholine occupied a comparatively distinct position. These groupings provide evidence of molecular similarity and differentiation within the evaluated material, but should be interpreted as locus-specific relationships rather than definitive genome-wide genetic relationships (Sakar et al., 2016). Comparable SSR-based analyses have successfully resolved cultivar relationships and identified genetically contrasting accessions in olive germplasm (Carriero et al., 2002; Koehmstedt et al., 2011; Li et al., 2020).

The molecular grouping showed only partial correspondence with the morpho-pomological classification, reinforcing that phenotypic similarity does not necessarily reflect molecular relatedness. Such discordance is expected because morphological expression can be influenced by environmental conditions and genotype × environment interactions, whereas SSR markers assess heritable variation at specific genomic loci. Similar differences between morphological and molecular classifications have been reported in olive (Rotondi et al., 2003; Rao et al., 2009), highlighting the complementary value of phenotypic and molecular approaches for cultivar discrimination and germplasm characterization. Accordingly, the present molecular and phenotypic classifications are best considered complementary rather than interchangeable.

The observed molecular grouping also does not necessarily reflect present-day geographical provenance. Olive has a long history of domestication, selection, exchange of planting material and vegetative propagation, which can obscure simple correspondence between genetic similarity and geographical origin. Previous SSR studies have similarly reported genetic groupings that do not consistently follow geographical distribution (Carriero et al., 2002; Noormohammadi, 2009; Ercişli et al., 2011; Li et al., 2020). The relationships observed here may therefore reflect shared genetic backgrounds and cultivar histories rather than geographical proximity alone.

From a germplasm-management and breeding perspective, the detected molecular variation provides useful baseline information for identifying genetically contrasting material for further evaluation. Genetically divergent accessions may serve as candidates for parental evaluation when combined with complementary agronomic, fruit-quality and reproductive traits; however, genetic distance alone is insufficient to predict breeding performance. The molecular profiles may also support cultivar authentication and conservation, particularly where phenotypic descriptors do not provide unequivocal discrimination.

The present investigation provides a molecular baseline for the thirteen olive accessions evaluated under the temperate Himalayan conditions of Jammu and Kashmir, complementing regional studies that have largely emphasized morphological variability and cultivar performance (Kumar et al., 2020, 2021). Although only three polymorphic SSR loci were identified, they detected allelic variation and differentiated the evaluated germplasm, demonstrating their utility for preliminary cultivar discrimination and germplasm characterization. Nevertheless, the limited number of loci restricts inference about genome-wide genetic structure. Further analysis using a larger panel of highly polymorphic SSRs or high-throughput approaches such as SNP genotyping and genotyping-by-sequencing (GBS) would provide greater resolution of genetic relationships and diversity and strengthen the molecular basis for olive germplasm management and improvement in the region.

### 4.8 Implications for cultivar selection and breeding

Collectively, the results demonstrate substantial phenotypic variation among the evaluated olive germplasm, with fruit size-related traits, particularly fruit length, fruit diameter, fruit weight, and flesh-to-stone ratio, providing useful descriptors for cultivar characterization under the evaluated temperate Himalayan conditions. Accessions, such as S1, S2, and Carolea exhibited comparatively higher fruit weight and favorable flesh-to-stone characteristics, indicating their potential value for further evaluation under local production conditions. The observed phenotypic differentiation and clustering patterns are broadly consistent with previous studies highlighting the utility of morpho-pomological traits for olive cultivar characterization (Khadivi et al., 2022; Casilla García et al., 2025; Dehghan-Seresht et al., 2025).

The SSR analysis additionally revealed molecular variation among the evaluated germplasm, with the polymorphic loci providing useful information for cultivar discrimination and assessment of genetic relationships. However, phenotypic and molecular variation represent distinct components of cultivar diversity and should not be interpreted as direct evidence that the observed phenotypic differences are genetically determined. Similarly, the present data do not permit definitive inference of genetic divergence underlying phenotypic differentiation or prediction of breeding performance. The integration of morpho-pomological, multivariate, and molecular analyses therefore provides complementary information for cultivar characterization and germplasm documentation. Overall, the study establishes a baseline characterization of the evaluated olive germplasm under temperate Himalayan conditions, while larger molecular datasets, broader germplasm sampling, and multi-environment evaluations will be necessary to validate genetic divergence, heritability, adaptation, and breeding value.

## 5 Conclusion

The present study revealed considerable morpho-pomological variation among the olive germplasm evaluated under temperate north-western Himalayan conditions of Jammu and Kashmir. Fruit size-related traits, particularly fruit length, fruit diameter, fruit weight, and flesh-to-stone ratio, were important contributors to cultivar differentiation and may serve as useful descriptors for cultivar characterization and selection under the evaluated conditions. The observed phenotypic variation and distinct grouping patterns obtained through multivariate analyses provide a useful basis for identifying promising cultivars and selections with desirable fruit and vegetative characteristics.

SSR-based characterization using three polymorphic loci detected molecular variation among the evaluated germplasm and provided preliminary information for cultivar discrimination and assessment of genetic relationships. However, given the limited number of polymorphic markers, these molecular results should be regarded as preliminary rather than a comprehensive assessment of genetic diversity or divergence. The integration of morpho-pomological, multivariate, and SSR-based analyses therefore provides a useful baseline for characterization and germplasm management, while avoiding direct inference of genetic determinants of the observed phenotypic variation. Overall, the study provides baseline information on the phenotypic and molecular characteristics of the evaluated olive germplasm under temperate Himalayan conditions. Further validation using a larger number of polymorphic molecular markers, broader germplasm collections, and multi-environment evaluations will be necessary to more comprehensively resolve genetic relationships, assess heritable variation, and identify promising materials for future cultivar selection and breeding programs in the Himalayan region.

## Data Availability

The original contributions presented in the study are included in the article/**Supplementary Material**. Further inquiries can be directed to the corresponding author.
