# Supplementary material for: Unraveling olive germplasm diversity through integrated phenotypic and SSR-based characterization under temperate Himalayan conditions
Source: Front Plant Sci. 2026 Oct 27;17:1934949. doi: 10.3389/fpls.2026.1934949 (PMC13748414; doi:10.3389/fpls.2026.1934949)
Supplement: Supplementary file 1 [file Table1.docx]

| **Cultivar** | **FL (mm)** | **FD (mm)** | **SD (mm)** | **FW (g)** | **FSR** | **SW (g)** | **St D (mm)** | **NG** | **LL (mm)** | **LW (mm)** |
| --- | --- | --- | --- | --- | --- | --- | --- | --- | --- | --- |
| **Zaituna** | 20.57 ± 0.09^e^ | 15.27 ± 0.15^f^ | 1.01 ± 0.02^e^ | 3.63 ± 0.10^h^ | 4.57 ± 0.15^d^ | 0.65 ± 0.04^i^ | 7.85 ± 0.25^h^ | 11.33 ± 0.58^a^ | 75.53 ± 2.15^a^ | 14.27 ± 0.04^f^ |
| **Nocellara Messinese** | 21.11 ± 0.45^de^ | 13.87 ± 0.47^g^ | 0.83 ± 0.03^h^ | 2.69 ± 0.10^k^ | 3.24 ± 0.08^g^ | 0.63 ± 0.04^j^ | 9.29 ± 0.25^d^ | 7.67 ± 1.15^de^ | 65.43 ± 1.72^c^ | 15.02 ± 0.49^e^ |
| **Moraiolo** | 20.99 ± 0.46^e^ | 15.33 ± 0.32^f^ | 0.97 ± 0.02^f^ | 3.92 ± 0.13^f^ | 3.26 ± 0.05^g^ | 0.92 ± 0.04^e^ | 8.54 ± 0.25^g^ | 5.67 ± 0.58^f^ | 41.40 ± 1.75^f^ | 9.45 ± 0.23^g^ |
| **Picholine** | 27.02 ± 0.56^a^ | 16.97 ± 0.49^cd^ | 1.05 ± 0.02^cd^ | 3.75 ± 0.09^g^ | 2.16 ± 0.07^i^ | 1.19 ± 0.05^a^ | 10.20 ± 0.30^b^ | 7.67 ± 0.58^de^ | 52.53 ± 2.25^d^ | 14.02 ± 0.28^f^ |
| **Coratina** | 19.83 ± 0.30^f^ | 17.57 ± 0.58^c^ | 1.11 ± 0.02^b^ | 4.74 ± 0.15^d^ | 4.51 ± 0.06^d^ | 0.86 ± 0.04^g^ | 9.85 ± 0.25^c^ | 9.00 ± 1.00^bc^ | 77.00 ± 1.14^a^ | 15.95 ± 0.36^d^ |
| **Carolea** | 24.13 ± 0.44^c^ | 19.93 ± 0.51^a^ | 1.32 ± 0.03^a^ | 6.48 ± 0.20^b^ | 5.57 ± 0.07^b^ | 0.99 ± 0.04^c^ | 9.78 ± 0.28^c^ | 9.67 ± 0.58^b^ | 72.60 ± 1.91^b^ | 15.33 ± 0.68^de^ |
| **Itrana** | 20.80 ± 0.12^e^ | 16.50 ± 0.44^de^ | 1.03 ± 0.02^de^ | 4.22 ± 0.13^e^ | 4.73 ± 0.14^c^ | 0.74 ± 0.04^h^ | 10.70 ± 0.30^a^ | 7.33 ± 0.58^e^ | 52.67 ± 1.85^d^ | 8.60 ± 0.28^h^ |
| **Cornicabra Attica** | 17.40 ± 0.24^g^ | 16.07 ± 0.25^e^ | 1.06 ± 0.02^cd^ | 2.76 ± 0.13^j^ | 4.19 ± 0.11^e^ | 0.53 ± 0.04^k^ | 9.10 ± 0.25^e^ | 8.33 ± 0.58^cde^ | 53.67 ± 2.10^d^ | 18.47 ± 0.33^b^ |
| **Cerignola** | 16.80 ± 0.42^g^ | 13.90 ± 0.46^g^ | 0.75 ± 0.03^i^ | 2.13 ± 0.10^l^ | 3.33 ± 0.11^f^ | 0.49 ± 0.04^l^ | 7.20 ± 0.25^i^ | 8.67 ± 1.53^bcd^ | 45.47 ± 1.79^e^ | 14.08 ± 0.25^f^ |
| **Nocellara del Belice** | 21.68 ± 0.50^d^ | 16.23 ± 0.38^e^ | 1.00 ± 0.02^ef^ | 3.68 ± 0.09^h^ | 2.65 ± 0.07^h^ | 0.85 ± 0.04^g^ | 9.85 ± 0.25^c^ | 8.33 ± 0.58^cde^ | 77.03 ± 1.27^a^ | 19.13 ± 0.25^b^ |
| **Etnea** | 19.24 ± 0.09^f^ | 15.00 ± 0.62^f^ | 0.92 ± 0.02^g^ | 3.39 ± 0.10^i^ | 2.64 ± 0.07^h^ | 0.89 ± 0.04^f^ | 8.95 ± 0.25^f^ | 7.67 ± 0.58^de^ | 42.47 ± 1.90^f^ | 9.92 ± 0.45^g^ |
| **S1** | 26.08 ± 0.46^b^ | 18.70 ± 0.36^b^ | 1.05 ± 0.03^d^ | 7.95 ± 0.23^a^ | 6.06 ± 0.12^a^ | 1.13 ± 0.05^b^ | 10.73 ± 0.35^a^ | 11.33 ± 0.58^a^ | 41.67 ± 1.85^f^ | 16.90 ± 0.47^c^ |
| **S2** | 25.72 ± 0.49^b^ | 20.10 ± 0.30^a^ | 1.08 ± 0.02^bc^ | 6.23 ± 0.15^c^ | 5.52 ± 0.12^b^ | 0.96 ± 0.04^d^ | 6.72 ± 0.12^j^ | 11.33 ± 0.58^a^ | 53.37 ± 1.95^d^ | 22.99 ± 0.16^a^ |

**Supplementary Table 1**. Mean performance of thirteen olive cultivars for quantitative fruit, stone and leaf morphological traits expressed as mean ± standard error. Means followed by different letters within a column differ significantly according to Fisher’s LSD test at P ≤ 0.05.

**FL** = Fruit length (mm); **FD** = Fruit diameter (mm); **SD** = Stalk diameter (mm); **FW** = Fruit weight (g); **FSR** = Flesh-to-stone ratio; **SW** = Stone weight (g); StD = Stone Diameter (mm); **NG** = Number of grooves; **LL** = Leaf length (mm); **LW** = Leaf width (mm).

| **Trait** | **FL** | **FD** | **SD** | **FW** | **FSR** | **SW** | **StD** | **NG** | **LL** | **LW** |
| --- | --- | --- | --- | --- | --- | --- | --- | --- | --- | --- |
| **FL** | 1.000 | 0.685** | 0.483ns | 0.716** | 0.283ns | 0.847** | 0.274ns | 0.332ns | -0.016ns | 0.293ns |
| **FD** | 0.685** | 1.000 | 0.820** | 0.859** | 0.684** | 0.619* | 0.160ns | 0.521ns | 0.139ns | 0.488ns |
| **SD** | 0.483ns | 0.820** | 1.000 | 0.664* | 0.547ns | 0.511ns | 0.351ns | 0.305ns | 0.366ns | 0.252ns |
| **FW** | 0.716** | 0.859** | 0.664* | 1.000 | 0.764** | 0.699** | 0.280ns | 0.544ns | -0.028ns | 0.315ns |
| **FSR** | 0.283ns | 0.684** | 0.547ns | 0.764** | 1.000 | 0.105ns | 0.007ns | 0.657* | 0.090ns | 0.335ns |
| **SW** | 0.847** | 0.619* | 0.511ns | 0.699** | 0.105ns | 1.000 | 0.426ns | 0.109ns | -0.155ns | 0.056ns |
| **StD** | 0.274ns | 0.160ns | 0.351ns | 0.280ns | 0.007ns | 0.426ns | 1.000 | -0.222ns | 0.091ns | -0.264ns |
| **NG** | 0.332ns | 0.521ns | 0.305ns | 0.544ns | 0.657* | 0.109ns | -0.222ns | 1.000 | 0.250ns | 0.601* |
| **LL** | -0.016ns | 0.139ns | 0.366ns | -0.028ns | 0.090ns | -0.155ns | 0.091ns | 0.250ns | 1.000 | 0.332ns |
| **LW** | 0.293ns | 0.488ns | 0.252ns | 0.315ns | 0.335ns | 0.056ns | -0.264ns | 0.601* | 0.332ns | 1.000 |

**Supplementary Table 2.** Pearson’s correlation coefficients among ten quantitative morphological and pomological traits of thirteen olive cultivars.

FL = Fruit length; FD = Fruit diameter; SD = Stalk diameter; FW = Fruit weight; FSR = Flesh-to-stone ratio; SW = Stone weight; StD = Stone diameter; NG = Number of grooves; LL = Leaf length; LW = Leaf width.

ns = non-significant; * significant at **P ≤ 0.05**; ** significant at **P ≤ 0.01**.

**Supplementary Table 3.** Mean CIELAB colour coordinates (**L***, **a***, and **b***) of fruits from thirteen olive cultivars evaluated under temperate Himalayan conditions.

| **Cultivar** | **L*** | **a*** | **b*** |
| --- | --- | --- | --- |
| Zaituna | 8.87 | 5.53 | 1.43 |
| Nocellara Messinese | 22.70 | 4.00 | -3.40 |
| Moraiolo | 8.80 | 10.00 | 4.50 |
| Picholine | 62.53 | -15.53 | 46.03 |
| Coratina | 27.57 | 12.03 | 7.47 |
| Carolea | 63.53 | -17.90 | 42.90 |
| Itrana | 16.87 | 7.97 | -3.93 |
| Cornicabra Attica | 21.07 | 17.23 | 9.70 |
| Cerignola | 11.67 | 0.63 | -1.20 |
| Nocellara del Belice | 20.83 | 8.50 | 3.40 |
| Etnea | 23.97 | 4.53 | 1.00 |
| S1 | 26.53 | 3.50 | 1.97 |
| S2 | 13.20 | 3.03 | 1.30 |
